# Multidisciplinary clinical guidelines in proactive monitoring, early diagnosis, and effective management of trastuzumab deruxtecan (T-DXd)-induced interstitial lung disease (ILD) in breast cancer patients

**DOI:** 10.1016/j.esmoop.2023.102043

**Published:** 2023-11-10

**Authors:** D. Wekking, M. Porcu, B. Pellegrino, E. Lai, G. Mura, N. Denaro, L. Saba, A. Musolino, M. Scartozzi, C. Solinas

**Affiliations:** 1 Amsterdam UMC, Location Academic Medical Centre, University of Amsterdam, Amsterdam, The Netherlands; 2 Radiology Department, AOU Cagliari, Cagliari University, Policlinico di Monserrato, Monserrato (CA); 3 Department of Medicine and Surgery, University of Parma, Parma; 4 Medical Oncology and Breast Unit, University Hospital of Parma, Parma; 5 Gruppo Oncologico Italiano di Ricerca Clinica(GOIRC), Parma; 6 Medical Oncology, AOU Cagliari, Policlinico di Monserrato, Monserrato; 7 Anatomical Pathology, Valdes Laboratory, Cagliari; 8 IRCCS Fondazone Ca' Granda Policlinico Milano, SC Oncologia, Milan, Italy

**Keywords:** trastuzumab deruxtecan, interstitial lung disease, T-DXd-induced ILD, HER2-positive breast cancer, HER2-negative breast cancer, multidisciplinary guidance

## Abstract

Trastuzumab deruxtecan (T-DXd), a human epidermal growth factor receptor 2 (HER2)-directed antibody-drug conjugate (ADC), has altered the treatment landscape in breast cancer (BC), irrespective of the HR-receptor status. The use of the agent is increasing, despite the finding that exposure to T-DXd increases the risk of interstitial lung disease (ILD), particularly in BC patients. Although T-DXd-related ILD can be potentially severe and life-threatening, most low-grade cases can be treated safely using a multidisciplinary approach comprising early and accurate diagnosis, effective management, close monitoring, and the prompt administration of steroids. Additionally, increasing patients' education on ILD symptoms ensures close attention and enables prompt reporting, enhancing patient outcomes. It is recommended that predictive biomarkers are assessed in patients with risk factors for developing ILD. Currently, diagnostic criteria comprise newly identified pulmonary opacities, the relation of symptom onset to medication initiation, and the exclusion of other causes of ILD. The general condition of patients is weakened during the management of ILD (BC progression and corticosteroid treatment). Consequently, BC chemotherapy might be attenuated. This highlights the importance of preventing (high-grade) ILD, especially since its use is expanded. Identifying high-risk patients, diagnosing, and customizing treatment is, however, challenging and additional information on patient selection is often not fully clarified. In this paper, we provide updated multidisciplinary clinical guidance for patient selection, proactive monitoring, early diagnosis, and effectively management of T-DXd-induced ILD in HER2-positive BC patients. We describe the risk factors for developing ILD, patients’ characteristics of ILD, and the histopathological and radiographic characteristics of ILD, including real-world clinical practice reports. These recommendations provide a structured step-by-step approach for managing each suspected BC-related ILD grade.

## Highlights

• Identifying risk factors for developing ILD is crucial in assessing suitability for T-DXd therapy and managing ILD.
• Grade 4 ILD can appear as diffuse bilateral ground-glass opacities, local parenchymal consolidation or thickened interlobular.
• In cases of symptomatic ILD, treatment with T-DXd should be discontinued immediately.
• The five ‘S’ rules help enhance patient outcomes by reducing the severity of ILD and fatal outcomes.
• Implementing a supportive reference chest computed tomography image retrieval system with deep learning enhances the diagnostic ability.

## Introduction

Breast cancer (BC) is the most frequently diagnosed tumor in women worldwide.^1^ Approximately 17% of breast tumors exhibit human epidermal growth factor receptor 2 (HER2) overexpression or amplification, commonly associated with an aggressive clinical phenotype.^2^ Dual therapy composed of HER2-directed humanized monoclonal antibody treatment with trastuzumab and pertuzumab combined with chemotherapy is currently the first-line treatment for patients with metastatic HER2-positive BC.

Over the past decade, HER2-directed antibody-drug conjugates (ADCs) have revolutionized the treatment landscape of both early-stage and advanced HER2-positive and -negative BC. These drugs are unique in delivering cytotoxic agents to tumors expressing HER2 on their surface and have the potential to play a role in early-line clinical treatment. For instance, trastuzumab deruxtecan (T-DXd), a HER2-directed ADC composed of a potent chemotherapy agent (topoisomerase I inhibitor; DXd) linked to a humanized monoclonal antibody with the same amino acid sequence as trastuzumab.^3^

The Food and Drug Administration (FDA) and European Medicines Agency approved T-DXd for treating unresectable or metastatic HER2-positive BC who have been pretreated with two or more anti-HER2-based regimens.^3^^,^^4^ More recently, the FDA extended the approval of T-DXd to unresectable or metastatic HER2-low BC patients who have received prior chemotherapy or developed recurrence during chemotherapy.^5^ Despite the efficacy and tolerable therapeutic index of the drug, there is increasing concern regarding interstitial lung disease (ILD). Exposure to T-DXd causes alveolar inflammation and interstitial fibrosis presumably by direct cytotoxic and immune-mediated pulmonary injury, which increases the risk of life-threatening or fatal ILD.^6^ Especially in BC, administration of this drug is associated with a substantial risk of ILD.^7^

T-DXd yielded significant ILD/pneumonitis signals (reporting odds ratio 91.07, 95% confidence interval 76.04-109.07), and the severity appears dose-dependent and related to the cytotoxic payload in alveolar macrophages.^8^ In previously treated advanced HER2-positive BC patients, treatment-induced ILD was observed in 10%-20% (mostly grade 1 or 2), with mortality ranging from 1%-12%, posing significant clinical issues.^3^^,^^9^ The median onset time is 5.6 months, and 97% of ILD events occur within the first year of treatment.^10^

The weakened general condition of patients during ILD management (due to BC progression and corticosteroid treatment) may attenuate BC chemotherapy, highlighting the importance of preventing high-grade ILD.^11^ With the increased use of T-DXd, preventing high-grade ILD is thus crucial; however, identifying high-risk patients, diagnosing, and customizing treatment is challenging. Hence, guideline consensus from a multidisciplinary team is critical to optimize early detection, therapeutic index, and management.

In this review, we will provide updated multidisciplinary clinical guidance for patient screening, proactively monitoring, early diagnosing, and effectively managing T-DXd-induced ILD in HER2-positive BC patients. We describe the risk factors for developing ILD and report a real-world clinical practice. These recommendations provide a structured step-by-step approach for each suspected BC-related ILD grade.

## T-DXd-related ILD in HER2-low/positive breast cancer: between clinical trials and clinical practice

Across the DESTINY clinical program, T-DXd has demonstrated a consistent tolerable safety profile. In the phase II DESTINY-Breast01 study (NCT03248492), ILD of any grade was observed in 15.8% (*n* = 29) of HER2-positive advanced BC patients who received T-DXd, of whom 2.7% (*n* = 5) were classified as grade 5.^3^ During this study, a 9-month follow-up was carried out, and only three new cases of ILD were reported, suggesting that the rate of discontinuation or ILD did not increase for patients who remained on treatment for a longer duration. The phase III DESTINY-Breast02 (NCT03523585) trial enrolled unresectable/metastatic HER2-positive BC patients previously treated with trastuzumab emtansine and observed 10.5% (*n* = 42) cases of T-DXd-related ILD. Among this group, 88.1% were reported as grade 1 or 2 cases and 0.5% as grade 5.^12^ Moreover, the phase III DESTINY-Breast03 (NCT03529110) trial reported (5.44 mg/kg three-weekly (q3w) and largely second-line) T-DXd-related ILD in 15.2% (*n* = 39) of previously treated HER2-positive metastatic BC patients. In this trial, no grade 4 or 5 cases were observed.^13^, ^14^, ^15^ Interestingly, longer follow-ups and increased treatment exposure led to an increase in ILD; however, compared with other clinical trials investigating metastatic BC, the new cases were reported as low-grade (1 or 2) and, thus, the rates of grade 3 or higher ILD were lower.^3^^,^^10^ The absence of ILD treatment-related deaths suggests that heavily pre-treated patients may be at higher risk for severe ILD. Moreover, increased awareness, earlier detection, implementation of management guidelines, and customized treatment could have contributed to this absence as well.

In clinical trials, T-DXd has been used to treat not only HER2-positive but also HER2-low BC patients. In the DESTINY-Breast04 phase III (NCT03734029) trial, 45 HER2-low advanced BC patients were treated with T-DXd, and T-DXd-related ILD occurred in 12.1% (10% grade 1 or 2, 1.3% grade 3 or 4, and 0.8% grade 5).^16^ A survey analysis was carried out among oncologists to investigate their perceptions of T-DXd-related ILD in HER2-low BC patients.^17^ Interestingly, 31% of oncologists documented fewer ILD cases in clinical practice than in the trial; however, 36% mentioned that ILD could be hard to quantify because of asymptomatic cases. Nevertheless, 60.2% of oncologist did not consider ILD a limiting factor in the use of T-DXd.

Recently, two studies have evaluated T-DXd-induced ILD: phase 1b/2 DESTINY-Breast07 (NCT04538742) in advanced or metastatic HER2-positive BC patients and phase 1 b DESTINY-Breast08 (NCT04556773) in HER2-low BC. To date, no cases of ILD have been reported.^18^ These results reinforce the consistent safety profile in BC patients observed in prior studies.

It is important to note that patients with a medical history of clinically relevant lung disease are often excluded from participating in trials; the study population may therefore be inconsistent with real-world clinical practice. It is reasonable to suggest that the toxicity experience is worse in clinical practice. Additionally, as aforementioned, T-DXd use is preferred even though ILD can be challenging to identify and manage. The incidence of ILD is therefore of clinical significance, emphasizing the importance of a risk-management approach with close monitoring of developing ILD-related adverse events.

## Patient eligibility for T-DXd and ILD characteristics

In order to minimize the likelihood of fatal outcomes associated with T-DXd treatment, it is crucial to conduct a comprehensive evaluation of the eligibility of patients for T-DXd treatment and carefully assess their ILD characteristics.

### Identify risk factors and features of interest

Prior to starting T-DXd therapy, physicians should conduct a comprehensive assessment of the ILD risk for each patient. Although the causality between risk factors for developing ILD and truly developing ILD remains uncertain, health providers should thoroughly evaluate the medical history and medication use of patients during the clinical examination, in order to identify potential risk factors.

Japanese ethnicity has been identified as a potential risk factor for ILD.^7^^,^^19^ The proportion of ILD cases was higher in patients of Japanese origin (24.4%) compared with non-Japanese (11%). One reason for this is the increased genetic susceptibility to ILD in this population. Conversely, ILD grade 3 or higher occurred regardless of race or country of origin.^20^ In addition to ethnicity, a high dose of T-DXd has also been identified as a possible risk factor for ILD.^20^^,^^21^ The standard dose of T-DXd is 5.4 mg/kg once every 3 weeks, which is considered to have a more positive benefit-risk profile compared with higher doses. Importantly, no meaningful reduction in efficacy was observed at the standard dose, and fewer safety events were reported.

Some clinical patient characteristics have been identified as predicting mortality in patients with ILD, e.g. acute and severe initial disease presentation, pre-existing ILD, hypoxemia, need for mechanical ventilation, and being elderly.^10^^,^^22^^,^^23^ Other features of interest include smoking, baseline SpO2 <95%, moderate/severe renal impairment at baseline, time since initial diagnosis of ≥3.9 years, and comorbidities. Comorbidities include pre-existing lung diseases (e.g. chronic obstructive pulmonary disease and asthma), use of drugs associated with high-ILD-related mortality, non-small-cell lung cancer, unilateral pneumonectomy, underlying rheumatologic or auto-immune disorders, and thus pre-existing ILD, which need to be confirmed/ruled out prior to starting therapy because of their higher risk of ILD.^10^^,^^24^

Importantly, it remains unclear whether patients with prior other treatment-related pneumonitis are at higher risk of T-DXd-included ILD, as the mechanisms of action of these toxicities appear to be different from those of T-DXd. For instance, patients with HER2-low disease may have received prior therapies associated with pneumonitis, such as everolimus and CDK4/6 inhibitors.

Overall, a detailed evaluation of each patient’s risk factors is critical in assessing their suitability for T-DXd therapy, as exclusion from treatment with this agent is generally not warranted even in patients with the aforementioned risk factors. Moreover, identifying risk factors is important in managing ILD which will be discussed in more detail later in this paper. Patients with one or more risk factors may require corticosteroids when grade 1 ILD is suspected or confirmed.

### Radiological and histopathological features of ILD

In the management of ILD, radiological techniques play a critical role in appropriate monitoring and diagnosis. Among these techniques, high-resolution computed tomography (CT) chest protocols are particularly valuable in assessing the patterns of ILD and grading the extent of lung involvement.^6^^,^^25^ During CT scans, a variety of radiological findings associated with drug-induced ILD can be observed and classified into different patterns. For instance, organizing pneumonia patterns [e.g. organizing pneumonia (OP) and chronic eosinophilic pneumonia], non-specific interstitial pneumonia (NSIP) patterns (e.g. NSIP and interstitial pneumonia), hypersensitivity pneumonitis patterns (e.g. non-fibrotic hypersensitivity pneumonia), diffuse alveolar damage (DAD) patterns (e.g. DAD, and acute interstitial pneumonia), simple pulmonary eosinophilia patterns (e.g. pulmonary eosinophilia), and other patterns such as sarcoid-like granulomatosis, pneumonitis flare and radiation recall pneumonitis (Table 1).^26^ Differentiating between these patterns contributes to accurate diagnosis and could enhance treatment outcomes.

**Table 1:** Histological and radiological features of drug-related interstitial lung disease (ILD). Patterns of drug-induced ILD are described based on their histological features and most common radiological findings. ILD grade and occurrence is given^6^^,^^25^^,^^26^

Patterns of drug-induced ILD	Histological features	Most common radiological findings	Median grade and occurrence
Organizing pneumonia	- Intra-alveolar, intra-bronchial granulation tissue - Interstitial inflammation - Can progress to diffuse alveolar damage (DAD)	- Bilateral, multifocal, peripheral, and peribronchovascular ground-glass opacities (GGOs) and/or consolidation areas with predominance in the mid-lower lung - Eosinophilic pneumonia peripheral band-like opacities and predominance in the upper lobes	Grade 2 Most common form
Non-specific interstitial pneumonia	- Thickening of the pulmonary interstitium due to an inflammatory infiltrate	- Bilateral, patchy, or diffuse GGOs with or without reticular opacities with peripheral and basilar predominance - Lung fibrosis shows temporal and spatial homogeneity, and lung abnormalities are usually symmetrical and bilateral	Grade 1 Second most common form
Hypersensitivity pneumonitis	- Cellular bronchiolitis, granulomas, multinucleated giant cells, interstitial inflammation	- Areas of GGOs with or without centrilobular lung nodules may be diffuse or predominantly distributed in the upper lobes - Air trapping	Grade 1 Rare
DAD	- Acute phase characterized by exudative alveolar edema, necrosis of type II pneumocytes and alveolar endothelial cells, and the presence of hyaline membranes and inflammation - Subacute phase by fibrosis (especially at levels of alveolar septa) and hyperplasia of type II pneumocytes	- GGOs or dependent consolidation in the majority of the lung are usually observed - Crazy paving = interlobular septal thickening and intralobular lines	Grade 3/4 Associated with acute clinical symptom presentation
Simple pulmonary eosinophilia		- Unilateral or bilateral non-segmental patchy GGOs or areas of consolidation	Grade 1 Associated with asymptomatic presentation

**Figure 1:**
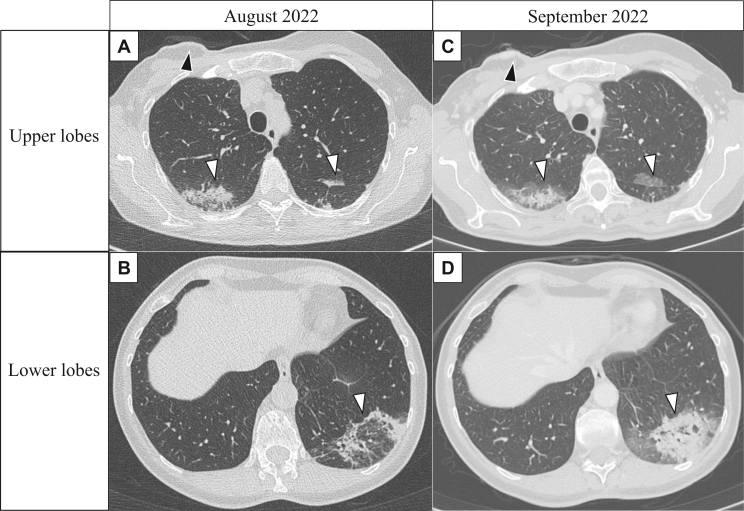
**Example of interstitial lung disease induced by trastuzumab deruxtecan therapy in a 53-year-old female patient with T4 breast cancer at the Parma Hospital**. The images show patterns of interstitial lung disease evidenced in a non-contrast computed tomography (CT) scan carried out in August 2022 (A and B), radiologically worsened in a subsequent contrast enhanced CT scan carried out 1 month later in September 2022 (C and D). A cryptogenic organizing pneumonia (COP) pattern characterized by ground-glass opacities and lung consolidations (white arrowheads) can be observed in the most dependent portions of both the upper lobes (A and C) and in the posterior basal segment of the left lower lobe. In the upper lobes (A and B) it is also possible to observe the dimensional growth of the breast cancer located in the right breast, associated with skin involvement.

The most common pattern of drug-induced ILD observed during imaging is OP characterized by ground-glass opacities (GGOs) and/or consolidations. Figure 1 shows an example CT images of a patient with T4 breast cancer who developed ILD induced by T-DXd. Follow-up CT scan 1 month later show worsening radiographic findings.

**Figure 2:**
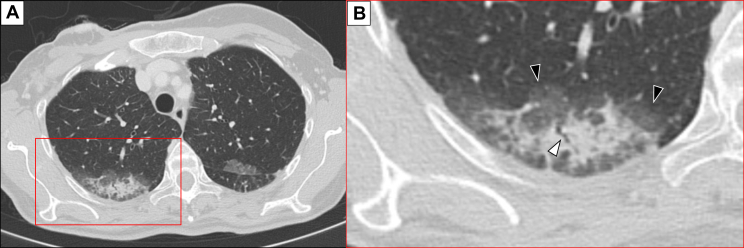
Same patient as in Figure 1, who experienced interstitial lung disease induced by trastuzumab deruxtecan therapy. (A) Lung consolidation (red square in A) seen in the right upper lobe of the non-contrast computed tomography scan carried out in August 2022. (B) Detail of the same lung consolidation, in which it is possible to observe the presence of air bronchogram within the consolidation (white arrowhead) and ground glass opacities (black arrowheads) around it.

In lung consolidations, formation of air bronchograms can be observed. Figure 2 shows a detailed CT scan of the same patient in which the presence of air bronchogram within the consolidation can be observed surrounded by GGOs. During differential diagnosis, the shape and luminal features of bronchi exhibiting the air bronchogram sign can assist in distinguishing between lung cancer, tuberculosis, and pneumonia.^27^ Furthermore, the extent of bronchial involvement displaying the air bronchogram sign, along with the ratio between the length of the affected bronchus and the size of the lesion, can be employed to differentiate lung cancer from tuberculosis and pneumonia.

The consolidations and GGO patterns during CT imaging are observed in other studies as well. One study reported specific CT findings of T-DXd-induced ILD in a patient with metastatic BC, which predominantly showed an OP pattern comprising subpleural consolidation in the lung periphery.^28^ Another study by Gocho et al. detected diffuse consolidation, thickening of the interlobular septum with GGO, pleural effusion, and reticular shadow on CT scans in patients with T-DXd-induced ILD in advanced BC.^11^ The presence of these findings should arouse suspicion for ILD, and immediate next steps should be taken if high-grade ILD is confirmed. It is also important to distinguish active ILD and fibrotic changes during resolution and align the CT findings with the clinical presentation. Supplementary Table S1, available at https://doi.org/10.1016/j.esmoop.2023.102043 provides an overview of common CT findings in patients with high grade ILD.

## Monitoring and diagnosing ILD during T-DXd therapy

### Importance of patients and health care provider education on ILD

In an effort to improve the management of ILD, it is important to prioritize safety, awareness, and proactive identification and monitoring. Adequate education for both health care providers and patients is crucial to facilitate the immediate reporting of signs and symptoms.^29^ Educate on the following symptoms: cough, fever, chest pain, dyspnea when walking, pleural effusion, and decreased SpO2, as well as non-specific symptoms such as fatigue, fever, and decreased appetite. Patients should be provided with a drug-specific guide for ILD and offered dedicated nurse-led informational classes before treatment. It is important to raise awareness of whom to contact and how for reporting early signs and symptoms.

Advise patients on the risk factors for ILD and instruct patients to immediately report new or worsening symptoms of ILD. Create awareness of slow or acute presentation for non-specific symptoms: fatigue, fever, and decreased appetite. Notably, patients may even be asymptomatic.

Provider education regarding CT features of ILD are provided in the previous section. Other information to facilitate early detection and diagnosis is described in the following paragraph. A multidisciplinary approach with consultations with a medical oncologist, specialist nurse, pulmonologist, thoracic surgeon, pathologist, infectious disease specialist, and radiologist is advised for an accurate diagnosis.

### Proactive monitoring and confirmation

During follow-up visits, a comprehensive evaluation is necessary for effective management of T-DXd-induced ILD. Careful patient history taking, physical examination, and measurement of vital signs should be conducted, along with laboratory tests, CT chest scan, and pulmonary function testing with spirometry and diffusing lung capacity for carbon monoxide (DLCO). Bronchoscopy and bronchoalveolar lavage with or without transbronchial lung biopsy may also be relevant components of the evaluation process.^22^^,^^30^^,^^31^ A pulmonary consult is advised if DLCO is >10%. Laboratory tests should include complete blood count, arterial blood gases, liver and kidney function, electrolytes, CRP, erythrocyte sedimentation rate, procalcitonin, LDH, with or without KL-6, surfactant protein A (SP-A), and SP-D. In addition, based on clinical suspicion, blood culture, sputum, and urinary antigens should be analyzed, along with the assessment of tumor markers and auto-immune antibodies to exclude any other cause of ILD.

Recent technological advancements have led to the use of exhaled breath analysis in diagnosing ILD, specifically through eNose technology, which has shown promise in accurately identifying the grade of ILD and serving as a possible biomarker at an earlier stage.^32^ In a study by Chang et al., induced ILD via anti-neutrophil cytoplasmic autoantibodies (ANCA) was reported, and testing for ANCA levels may be considered in BC patients at high risk of developing ILD prior to or during T-DXd treatment.^33^ Additionally, anti-PR3 has been found to be correlated with the Birmingham vasculitis score during corticosteroid treatment, although this correlation was lost after salvage treatment with rituximab. During this treatment, myeloperoxidase-DNA demonstrated a better correlation with clinical severity. myeloperoxidase-DNA has been identified as a promising biomarker for monitoring vasculitis activity in ILD, as it is a biomarker of neutrophil extracellular traps. Arakawa et al. revealed stratifin as a biomarker for drug-induced ILD via diffuse alveolar damage, and testing stratifin in serum can distinguish diffuse alveolar damage from other histopathological patterns of ILD or other lung diseases.^31^ Another study identified a biomarker to differentiate drug-induced ILD from other lung diseases and ILD grades through testing LPC(14:0).^34^ While these tests may be useful in specific patients, further research is needed to determine their role in clinical follow-up visits.

**Figure 3:**
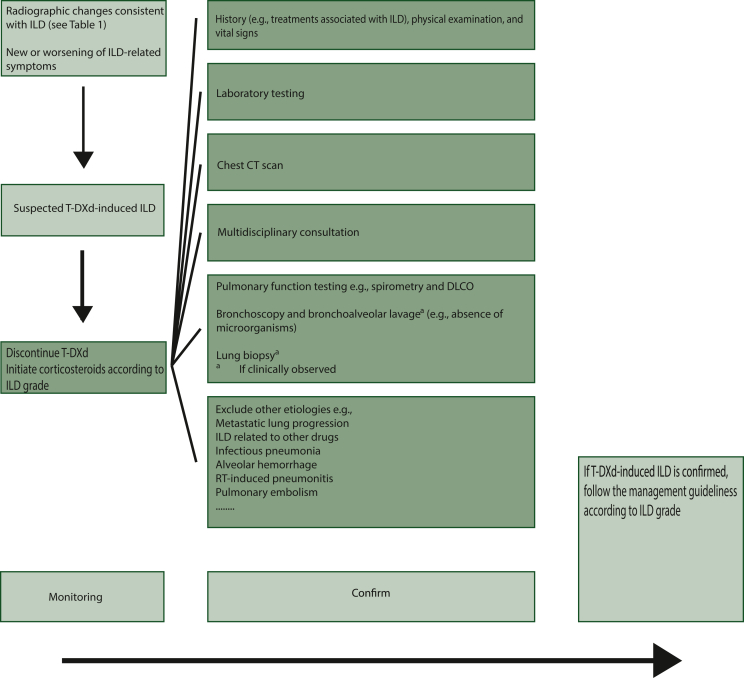
**Recommendations for the multidisciplinary monitoring and diagnosis of T-DXd-induced ILD based on DESTINY-Breast03 and DESTINY-Breast04 Protocols.**^14^^,^^16^ Careful patient history taking, physical examination, vital sign measurement, laboratory tests, CT chest scan every 9-12 weeks or when new symptoms appear, pulmonary function testing with spirometry and diffusing lung capacity for carbon monoxide (DLCO) are all relevant. A laboratory test should include complete blood count, arterial blood gases, liver and kidney function, electrolytes, CRP, erythrocyte sedimentation rate, procalcitonin, LDH, with or without KL-6, surfactant protein A (SP-A), and SP-D. Based on clinical suspicion, blood culture, sputum, and urinary antigens should be analyzed, as well as assessing tumor markers and autoimmune antibodies. Exclude any other cause of ILD. If necessary, BAL and lung biopsy might be helpful. BAL, bronchoalveolar lavage; CT, computed tomography; ILD, interstitial lung disease; RT, radiotherapy; T-DXd, trastuzumab deruxtecan.

In the context of ILD, monitoring and confirmation are crucial components of disease management. High-resolution CT scan is recommended at intervals of either 6 weeks in clinical trials or 9-12 weeks in clinical practice, or when new respiratory symptoms arise. In cases where ILD is suspected (developing new/worsening pulmonary or other related signs/symptoms and/or if a patient develops radiographic changes potentially consistent with ILD) , T-DXd therapy should be interrupted and ILD should be ruled out by means of various diagnostic measures, including monitoring of DLCO (Figure 3). Monitoring of DLCO is a reasonable method for assessing the onset of ILD. One study suggests that stable DLCO values may indicate the absence of ILD onset.^35^ The feasibility of utilizing chest imaging and pulmonary function testing, including DLCO measurement, for monitoring HER2+ or HER2-low metastatic BC patients receiving T-DXd has been investigated, with promising results indicating that such monitoring may prevent delays in treatment for patients suspected of having ILD.

In addition, it is recommended that bronchoalveolar lavage be considered to exclude infectious pneumonia, alveolar hemorrhage, and metastatic tumor colonization when the differential diagnosis is inconclusive or when there is no clinical improvement after attenuation of T-DXd therapy.^25^ Furthermore, other potential causes of ILD such as pulmonary embolisms, radiation-induced pneumonitis, cardiopathy, and lymphangitic carcinomatosis should be ruled out.^36^ If the diagnosis remains uncertain after the use of the aforementioned diagnostic tools, a lung biopsy is suggested. The pathologist can identify characteristic histological features to aid in making the diagnosis.

It is crucial to closely monitor patients to exclude any other potential causes of ILD. If the diagnosis of ILD is uncertain, referral to a pulmonologist is recommended prior to attenuating T-DXd therapy and initiating corticosteroids. When assessing the benefit-risk ratio of T-DXd treatment, compromised lung function or its exclusion should be taken into consideration. The pulmonologist can predict the drug benefit-risk profile for a specific patient based on exposure-response, exposure-safety, pharmacokinetic analysis, and study data. The recommended dose of T-DXd will be identified, taking into account the patient's medical history, differential diagnosis, and contraindications (Supplementary Table S2, available at https://doi.org/10.1016/j.esmoop.2023.102043). While a definitive diagnosis is not always necessary, early treatment (initiating corticosteroids and attenuating T-DXd) is critical for improved patient outcomes. If another etiology for ILD cannot be identified, the ILD management guidelines should be followed.

## Recommended multidisciplinary management of T-DXd-associated ILD

**Figure 4:**
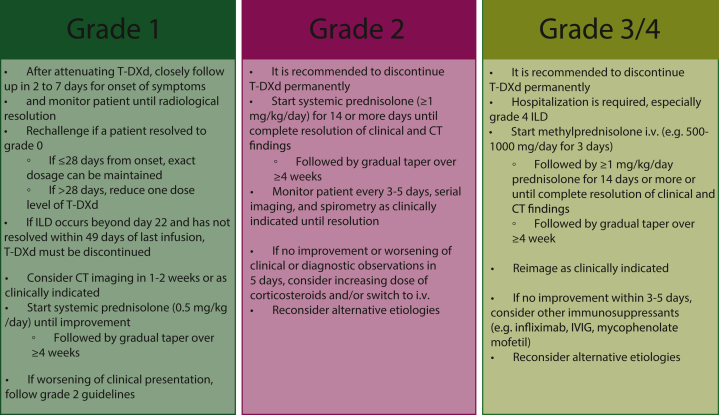
**Recommended multidisciplinary management of T-DXd-associated ILD based on DESTINY-Breast03 and DESTINY-Breast04 Protocols.**^14^^,^^16^ ILD, interstitial lung disease; IVIG, intravenous immunoglobulin; T-DXd, trastuzumab deruxtecan.

Management of ILD is a complex process that involves suppressing inflammation and preventing fibrosis. Consensus guidelines are needed for ILD monitoring, diagnosis, and management. The severity of ILD dictates the appropriate treatment approach. In cases where ILD is confirmed, multidisciplinary management is recommended, which includes the involvement of various healthcare professionals including pulmonologists, oncologists, radiologists, and pathologists.^29^ In this section, recommendations will be given based on the DESTINY-Breast03 and DESTINY-Breast04 Protocols (Figure 4).^14^^,^^16^

### Grade 1

Grade 1 is defined as asymptomatic with radiographic findings only. It is recommended to temporarily discontinue T-DXd and monitor patients until radiological resolution occurs.^3^^,^^13^^,^^15^^,^^16^ Rechallenge with T-DXd is only recommended for patients who resolve to grade 0 ILD. If resolution occurs within 28 days of onset, the exact T-DXd dosage can be maintained. However, if resolution takes longer, one dose level reduction is advised based on DESTINY-Breast03 and DESTINY-Breast04 Protocols, such as reducing the starting dose of 5.4 mg/kg q3w to 4.4 mg/kg q3w.^37^ The second-level reduction is 3.2 mg/kg q3w. Discontinuation of T-DXd is recommended if further reductions are needed or if ILD occurs beyond day 22 and has not resolved within 49 days of the last infusion.

Close monitoring and follow-up visits within 1 week after discontinuing T-DXd are necessary to evaluate for onset clinical symptoms and pulse oximetry. Consider CT imaging in 1-2 weeks or as clinically indicated. Steroid treatment may be warranted for some patients grade 1 to help mitigate progression to higher-grade. It is advised to select grade 1 cases that show extensive lung involvement or those at higher risk for progression to a higher grade. A systemic prednisolone regimen (0.5 mg/kg/day) is recommended until improvement, followed by gradual tapering over ≥4 weeks. Although the prognosis for grade 1 ILD patients is generally favorable, if diagnostic observations worsen despite the initiation of corticosteroids, grade 2 guidelines should be followed.

### Grade 2

Mild respiratory symptoms define grade 2. For patients in this category, it is recommended to permanently discontinue T-DXd.^3^^,^^13^^,^^15^^,^^16^ Treatment with systemic prednisolone at a dose of ≥1 mg/kg/day for ≥14 days is recommended until complete resolution of clinical and chest CT findings, followed by a gradual taper over ≥4 weeks. Care should be taken during the tapering process, as rapid tapering may increase the risk of ILD reactivation and adverse events associated with long-term corticosteroid use. It is advised to closely monitor clinical condition every 3-5 days until resolution, along with serial imaging and spirometry as clinically indicated. If there is no improvement in clinical or diagnostic observations or worsening of the general condition within 5 days, consider increasing the dose of corticosteroids (2 mg/kg/day) and switching to intravenous administration. It is important to reconsider additional workup for alternative etiologies.

### Grades 3 and 4

Grade 3 ILD is characterized by symptoms that impair quality of life and restrict daily activities, while grade 4 ILD is defined by severe, disabling symptoms that may require mechanical ventilation. In these cases, it is advised to permanently discontinue T-DXd.^3^^,^^13^^,^^15^^,^^16^ Hospitalization is required, especially for grade 4 cases. High-dose methylprednisolone should be promptly administered intravenously (e.g. 500-1000 mg/day for 3 days), followed by prednisone at a dose of ≥1 mg/kg/day for 14 days or until complete resolution of clinical and chest CT findings. Subsequently, the dosage should be gradually tapered over ≥4 weeks, and re-imaging should be carried out as clinically indicated. If there is no improvement within 5 days, alternative etiologies should be considered, and additional immunosuppressants (e.g. infliximab, intravenous immunoglobulin, mycophenolate mofetil) should be considered according to local practice.

It is important to provide supportive treatment for prolonged corticosteroid use, and steroid tapering should be done slowly to prevent ILD reactivation. It is crucial to avoid pulmonary function tests during the acute phase of grade 3 or higher and delay them until improvement is observed.^6^ Resolution of ILD is achieved when ground-glass opacities (GGOs) with residual fibrotic alterations without progression of other components are resolved, and clinical presentation is improved.^6^

### Applicability and contra-indications of corticosteroid treatment

In the context of systemic corticosteroid administration, careful observation is paramount, particularly in elderly patients. The well-known side effects of the medication including osteoporosis, hypertension, hypokalemia, diabetes, increased susceptibility to infections, and skin atrophy, can potentially result in life-threatening situations.^38^^,^^39^ The clinical response should therefore be closely monitored in elderly patients with low body weight, and the dose should be adjusted accordingly if necessary.

In addition, caution is advised during corticosteroid treatment in patients with renal failure because the drug is mainly excreted in the urine, and the concurrent use of loop diuretics and thiazide diuretics may increase the risk of hyperkalemia.^38^ Furthermore, the agent should be used carefully in patients with hepatic impairment. For instance, liver cirrhosis may alter the effects of prednisone and cortisone since these agents undergo conversion in the liver. Prednisolone and hydrocortisone are therefore recommended.

Patients with existing cardiovascular risk factors require monitoring for adverse events such as dyslipidemia and hypertension, especially during long-term corticosteroid use.^38^ If the expected duration of use is more than three months, osteoporosis prophylaxis and bone density scanning are recommended. Moreover, patients with risk factors for gastric complications should receive a gastric protector.

Corticosteroids are contraindicated in patients with underlying acute infections, particularly viral and systemic fungal infections.^38^^,^^39^ Extra caution is warranted when administering corticosteroids simultaneously with enzyme inducers or inhibitors, such as antibiotics. These agents can increase plasma concentration and co-administration may result in enhanced adverse events associated with corticosteroids.

## Future directions

Early and accurate diagnosis of T-DXd-induced ILD remains challenging despite the known risk factors. Evidence of the relation of these risk factors to the severity, and therefore the benefit-risk ratio of T-DXd therapy, is, however, scarce. Although Japanese ethnicity has been identified as a factor of interest for developing ILD, it does not necessarily indicate a higher risk of fatal outcomes. ILD grade ≥3 occurred regardless of race or country of origin. In general, none of the known risk factors are considered contraindications for T-DXd therapy, except for pre-existing severe dyspnea, hypersensitivity to T-DXd and active radiographic findings. Further research is needed to establish a relationship between these factors and the risk of T-DXd-induced ILD.

The eNose technology, breath analysis, has shown promise in accurately identifying the grade of ILD and might therefore predict fatal outcomes as well as testing LPC(14:0). Further investigations are crucial to identify more biomarkers predicting or detecting (high-risk) ILD. It is important to note that upregulation of the aforementioned biomarkers is not observed in all cases of ILD, and their absence does not necessarily indicate the absence of ILD. Additionally, further research is required to determine whether these biomarkers can truly prevent clinically fulminant outcomes.

Digital health tools like portable SpO2 monitoring could enhance the early detection of T-DXd-related ILD. Another way to enhance diagnostic ability is to implement a supportive reference chest CT image retrieval system with deep learning with top differential diagnoses and definitive diagnoses.^40^ This way, a definite diagnosis can be made based on the perception and experience of the physician, enriched and supported by an artificial intelligence algorithm. For widespread applicability, this database should continuously be updated with more and different reference ILD cases, incorporating other novel patterns of ILD. The management of T-DXd-induced ILD could also be improved with future studies focusing on the validity of rechallenging asymptomatic patients that have not resolved to grade 0 or those with fully resolved grade 2 ILD.

In summary, early and accurate diagnosis of T-DXd-induced ILD is crucial in ensuring favorable patient outcomes. Continued research in identifying predictive biomarkers, implementing digital health tools, and improving management strategies is essential for enhancing the diagnosis and management of T-DXd-induced ILD.

## Conclusion

T-DX-d has altered the treatment landscape in BC patients, irrespective of the HR-receptor status. The overall clinical data support the favorable benefit-risk profile of T-DXd. Although T-DXd-related ILD can be potentially severe and life-threatening, the majority of low-grade cases can be safely managed through a comprehensive multidisciplinary approach that involves early and accurately diagnosis, prompt initiation of steroids, and close monitoring. Additionally, increasing patient education ensures close attention to their symptoms. Tarantino et al. summarized these strategies via the five ‘S’ rules including: screen, scan, synergy, suspend treatment, and steroids.^41^ Recent clinical trials have reported no or a low number of ILD cases, indicating that the ‘S’ rules help enhance patient outcomes by reducing the severity of ILD and fatal outcomes.

At present, there are no established guidelines for managing high-risk patients who may be more susceptible to developing severe ILD while receiving T-DXd treatment. It remains unclear whether specific risk factors, such as Japanese ethnicity, acute and severe initial disease presentation, pre-existing ILD or other lung diseases, hypoxemia, need for mechanical ventilation, advanced age, T-DXd dose over 5.4 mg/kg, moderate/severe renal impairment at baseline, smoking history, use of drugs associated with high-ILD-related mortality, non-small-cell lung cancer, unilateral pneumonectomy, underlying rheumatologic or autoimmune disorders, thoracic irradiation, and pre-existing ILD, truly contribute to a higher risk of developing high-grade ILD. It may therefore be prudent to test for predictive biomarkers in these patient populations both prior and during T-DXd treatment. There is, however, a current lack of such predictive biomarkers and further research is essential to provide new insights in this area.

In order to diagnose ILD associated with treatment using T-DXd, the current criteria includes identifying new pulmonary opacities, a temporal relationship with medication initiation, and exclusion of other causes of ILD. It is important for both patients and healthcare providers to be aware that symptoms may be non-specific and can present slowly or acutely. A multidisciplinary approach involving various specialists such as medical oncologists, specialist nurses, pulmonologists, thoracic surgeons, pathologists, infectious disease specialists, and radiologists is crucial in managing this condition.

In cases of symptomatic ILD, treatment with T-DXd should be discontinued immediately. Reintroduction of the medication can be considered in asymptomatic patients only after complete resolution, with dose reductions as needed. The cornerstone of ILD treatment is corticosteroid intervention, with dosing adjusted according to the severity of the adverse event. For grade 1 cases, it is advised to only treat patients with extensive lung involvement or patients at an increased risk for ILD progression. All patients with ILD, regardless of the grade, should continue to visit their healthcare providers until their condition is fully resolved. Customized treatment may improve the therapeutic index and allow for safe expansion of T-DXd treatment across various tumor types.
