# Supplementary material for: Multidisciplinary clinical guidelines in proactive monitoring, early diagnosis, and effective management of trastuzumab deruxtecan (T-DXd)-induced interstitial lung disease (ILD) in breast cancer patients
Source: ESMO Open. 2023 Nov 10;8(6):102043. doi: 10.1016/j.esmoop.2023.102043 (PMC10679891; doi:10.1016/j.esmoop.2023.102043)
Supplement: Supplementary Table S1 [file mmc1.docx]

**Supplementary Table S1.** Imaging features of drug-induced grade 4 ILD. Most common CT findings per phase of grade 4 drug-induced ILD are described ^6^.

| Phase in grade 4 ILD | Most common CT findings |
| --- | --- |
| Acute phase | Diffuse and bilateral GGOs, with areas of lobular sparing which can be associated with local parenchymal consolidation or thickened interlobular septa with crazy-paving patterns |
| Subacute phase | Peribronchial or subpleural opacity. A reversed halo sign could be observed |
| Chronic phase | Decreased consolidative opacities, with irregular reticulation and traction bronchiectasis characterize fibrosis |
| Resolution | Resolution of GGOs with residual fibrotic alteration without progression of other components |
