# Supplementary material for: Multidisciplinary clinical guidelines in proactive monitoring, early diagnosis, and effective management of trastuzumab deruxtecan (T-DXd)-induced interstitial lung disease (ILD) in breast cancer patients
Source: ESMO Open. 2023 Nov 10;8(6):102043. doi: 10.1016/j.esmoop.2023.102043 (PMC10679891; doi:10.1016/j.esmoop.2023.102043)
Supplement: Supplementary Table S2 [file mmc2.docx]

**Supplementary Table S2.** Differential diagnoses of drug-induced ILD patterns on radiological findings. Differential diagnosis per patter of drug-induced ILD are described as well as recommended additional tests ^26^.

| Patterns of drug-induced ILD | Differential Diagnosis |  | Additional tests |
| --- | --- | --- | --- |
| Organizing pneumonia (OP) | - Progression of malignancy - Infectious pneumonia - Chronic eosinophilic pneumonia - OP unrelated to T-DXd - Smoking-associated lung injury |  | - Lab compatible with infection  - microbial and serological testing  - BAL, lung biopsy |
| Non-specific interstitial pneumonia (NSIP) | - Infectious pneumonia - Interstitial pneumonia - NSIP associated with connective tissue disease |  | - Laboratory markers specific for connective tissue disease  - Lab compatible with infection  - microbial and serological testing  - BAL lung biopsy |
| Hypersensitivity pneumonitis (HP) | - Atypical infection - Respiratory bronchiolitis - Follicular bronchiolitis - Exposure-related HP - Smoking-associated lung injury |  | - Lab compatible with infection  - microbial and serological testing  - BAL lung biopsy |
| Diffuse alveolar damage (DAD) | - Infectious pneumonia - Pulmonary edema - Alveolar hemorrhage |  | - Laboratory test anemia  - Lab compatible with infection  - microbial and serological testing  - BAL lung biopsy |
| Simple pulmonary eosinophilia (SPE) | - Infectious pneumonia - Alveolar hemorrhage |  | - Laboratory test anemia  - Lab compatible with infection  - microbial and serological testing  - BAL lung biopsy |
| Sarcoid-like granulomatosis | - Progression of malignancy - Infection - Sarcoidosis |  | - Lab compatible with infection  - microbial and serological testing  - BAL lung biopsy |
| Pneumonitis flare | - Infectious pneumonia - OP unrelated to medication |  | - Lab compatible with infection  - microbial and serological testing  - BAL lung biopsy |
| Radiation recall | - Infection - Progression of malignancy |  | - Lab compatible with infection  - microbial and serological testing  - BAL lung biopsy |
